# Polymerization Shrinkage of Five Bulk-Fill Composite Resins in Comparison with a Conventional Composite Resin

**Published:** 2018-11

**Authors:** Mahdi Abbasi, Zohreh Moradi, Mansoureh Mirzaei, Mohammad Javad Kharazifard, Samaneh Rezaei

**Affiliations:** 1 Assistant Professor, Dental Research Center, Dentistry Research Institute, Tehran University of Medical Sciences, Tehran, Iran; Department of Restorative Dentistry, School of Dentistry, Tehran University of Medical Sciences, Tehran, Iran; 2 Assistant Professor, Department of Restorative Dentistry, School of Dentistry, Tehran University of Medical Sciences, Tehran, Iran; 3 Associate Professor, Department of Restorative Dentistry, School of Dentistry, Tehran University of Medical Sciences, Tehran, Iran; 4 Research Member, Dental Research Center, Dentistry Research Institute, Tehran University of Medical Sciences, Tehran, Iran; 5 Postgraduate Student, Department of Restorative Dentistry, School of Dentistry, Tehran University of Medical Sciences, Tehran, Iran

**Keywords:** Filtek Bulk Fill, Composite Resins, Polymerization

## Abstract

**Objectives:**

The polymerization shrinkage of methacrylate-based composites is among the most important causes of failure of composite restorations. The manufacturers claim that bulk-fill composites have a lower polymerization shrinkage than conventional composites. This study aimed to assess the polymerization shrinkage of five bulk-fill composites in comparison with a conventional composite.

**Materials and Methods:**

In this in-vitro experimental study, composite discs (n=30) were fabricated using everX Posterior (EXP), Filtek Bulk-Fill Posterior (FBP), SonicFill 2 (SF2), Tetric N-Ceram Bulk-Fill (TNB), X-tra fil (XF), and Filtek Z250 conventional composite at the center of a metal ring bonded to a microscope slide and were covered with a coverslip. This assembly was transferred to a linear variable differential transformer (LVDT). Light-curing (1200 mW/cm^2^) was performed from underneath the slide for 30 seconds. The deflecting disc method and LVDT were used to assess the dimensional changes of the samples (indicative of polymerization shrinkage) at 1, 30, 60, and 1800 seconds following the onset of light irradiation. Data were analyzed using one-way analysis of variance (ANOVA) and Tukey’s test.

**Results:**

The groups were significantly different regarding polymerization shrinkage (P<0.002). The polymerization shrinkage of the tested composites following the onset of light irradiation ranged from 0.19 to 3.03. EXP showed a significantly higher polymerization shrinkage than other composites at 30, 60, and 1800 seconds after light irradiation, while XF showed the lowest polymerization shrinkage at the aforementioned time points.

**Conclusions:**

The tested bulk-fill composites had a polymerization shrinkage similar to that of the conventional composite.

## INTRODUCTION

Composite resins are increasingly used for dental restoration due to their favorable features including low costs, conservative technique, and acceptable esthetics [1]. The physical and mechanical properties of composite resins have been greatly improved over the past couple of years; however, they still have some shortcomings [1]. Polymerization shrinkage is a common problem associated with light-cure composite resins [1,2].

In dental restoration, the dimensional stability of restorative materials plays an important role in the prevention of microleakage at the tooth-restoration interface [3]. The restorative material must remain dimensionally stable during polymerization and thermal and mechanical cycles. However, most composite resins do not meet this requirement, and their dimensional stability is influenced by the polymerization reactions of the matrix [3]. The polymerization shrinkage of composite resins occurs following the conversion of monomer molecules to a polymer structure through the replacement of van der Waals spaces with covalent bonds, leading to a decreased free volume [4]. The defects at the bonding interface are due to the polymerization shrinkage stress generated during restoration and subsequent thermal, functional, and mechanical stresses. The polymerization process and the magnitude of the volumetric shrinkage are influenced by the composition of the restorative material [4]. Stress generation is influenced by the reaction kinetics since a higher polymerization rate is associated with a greater polymerization stress. Moreover, there is a direct correlation between the increased amount of fillers and reduction of polymerization shrinkage. Thus, the addition of pre-polymerized resin fillers (organic fillers) decreases the volumetric reduction of polymerized resins and the consequent polymerization shrinkage [4]. The shrinkage stress can affect the marginal integrity and can result in marginal leakage, debonding, secondary caries, and postoperative tooth hypersensitivity [4–6]. Moreover, the curing stress can result in the formation of enamel microcracks and cuspal deflection in direct composite restorations with a high C-factor such as extensive Class I and mesiooccluso-distal (MOD) Class II cavities [4–6]. The magnitude of this shrinkage is influenced by factors such as the curing time, high intensities of the curing light, the matrix composition, the filler content, and the concentration of photo-initiators in composite resins [4–6].

Several methods have been proposed to minimize polymerization shrinkage including the incremental application of the composite, use of liners with a low modulus of elasticity as stress absorber, and soft-start polymerization [5]. Incremental application of composite resins has been suggested as a standard technique to decrease the polymerization shrinkage stress and to achieve an optimal degree of conversion. However, void formation, contamination, bond failure between the increments, and the time-consuming nature are among the drawbacks of this technique [5].

Considering the formulations of composite resins, light irradiation time may vary from 20 to 40 seconds for each increment, which is time-consuming and can cause patient dissatisfaction. However, despite the use of the incremental application technique, postoperative tooth hypersensitivity is still a common finding, which is mainly related to the polymerization shrinkage stress [5,7, 8].

Considering the shortcomings of conventional composite resins, bulk-fill composites were introduced to the market aiming to save time and lower the costs [9]. The main advantage of bulk-fill composites is their bulk application into the cavity with a thickness of up to 4 mm. Bulk-fill composites do not require an incremental application, longer curing times, or a higher light intensity for curing [9,10]. The manufacturers claim that bulk-fill composites have a polymerization shrinkage lower than that of flowable and conventional composites [11]. Bulk-fill composites have chemically altered monomers in their structure. The modifications made in the composition of the monomer and organic matrix of composites have resulted in over 70% reduction in the polymerization shrinkage stress [11].

Considering the relatively recent introduction of bulk-fill composites into the market, studies on the polymerization shrinkage of different brands of bulk-fill composites are limited. Thus, the present study aimed to assess the polymerization shrinkage of five bulk-fill composites in comparison with a conventional composite resin.

## MATERIALS AND METHODS

In this in-vitro experimental study, the sample size was calculated to be five samples in each of the six groups using one-way analysis of variance (ANOVA) and the power analysis feature of PASS II software (NCSS, LLC, Kaysville, UT, USA), assuming alpha=0.05, beta=0.2, standard deviation (SD)=0.04, and effect size=0.28 according to a study by Benetti et al [12]. The present study evaluated the polymerization shrinkage of five bulk-fill composites including everX Posterior (EXP), Filtek Bulk-Fill Posterior (FBP), SonicFill 2 (SF2), Tetric N-Ceram Bulk-Fill (TNB), and X-tra fil (XF), and one conventional composite, namely, Filtek Z250 as the control (n=5). Table 1 shows the characteristics of the composite resins used in the present study.

**Table 1.** Characteristics of the composite resins used in this study

** Code **	** Commercial brand **	** Type of composite **	** Manufacturer **	** Composition **	** Filler percentage **	** Color **
1	everX Posterior (EXP)	Short-fiber composite	GC Corp., Tokyo, Japan	Short E-glass fiber filler, barium glass, Bis-GMA, PMMA, TEGDMA	74.2 wt 53.6 vol	Universal
2	Filtek Bulk-Fill Posterior (FBP)	Nanofill	3M ESPE, St. Paul, MN, USA	Non-agglomerated/non-aggregated 20-nm silica filler, non-agglomerated/non-aggregated 4-nm to 11-nm zirconia filler, aggregated zirconia/silica cluster filler, ytterbium trifluoride filler consisting of agglomerate 100-nm particles, ERGP-DMA, diurethane-DMA, 1, 12-dodecane-DMA	76.5 wt 58.4 vol	A2
3	SonicFill 2 (SF2)	Nanohybrid	Kerr Corp., USA	Poly(oxy-1,2-ethanediyl), α,α′-[(1-methylethylidene)di-4, 1-phenylene]bis[ω-[(2- methyl-1-oxo-2-propen-1-yl)oxy], 2,2′-ethylenedioxydiethyl dimethacrylate	81.3 wt unreported	A2
4	Tetric N-Ceram Bulk-Fill (TNB)	Hybrid	Ivoclar Vivadent AG, Schaan, Liechtenstein, Germany	Barium glass, Prepolymer, Ytterbium trifluoride, Mixed oxide Bis-GMA, DMA	75–77 wt 53–55 vol	IVA
5	X-tra fil (XF)	Hybrid	VOCO, Cuxhaven, Germany	Barium-boron-aluminosilicate glass, Bis-GMA, UDMA, TEGDMA	86 wt 70.1 vol	Universal
6	Filtek Z250 Universal (Z250)	Microhybrid	3M ESPE, St. Paul, MN, USA	zirconia/silica without silane treatment, Bis-GMA, UDMA, Bis-EMA	82 wt 60 vol	A2

Bis-GMA=bisphenol A glycidyl methacrylate, PMMA=poly (methyl methacrylate), TEGDMA=triethylene glycol dimethacrylate, UDMA=urethane dimethacrylate, Bis-EMA= bisphenol A ethoxylate dimethacrylate

Polymerization shrinkage was evaluated using the bonded disc or the deflecting disc technique which evaluates the dimensional changes of the samples during polymerization using the linear variable differential transformer device (LVDT; RDP Electronics Ltd., Wolverhampton, UK). For this purpose, 0.2 mg of each composite resin, in the form of an uncured paste, was applied on a microscope slide measuring 1×25×75 mm^3^ at the center of a metal ring with the diameter of 16 mm and the height of 1.5 mm.

**Fig. 1:**
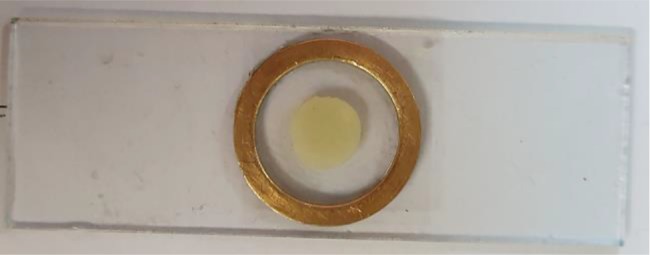
A sample of a packed composite resin

Another slide was compressed over the paste to pack the composite sample into the ring (Fig. 1). The surfaces of the sample and the metal ring were then covered with a coverslip (0.13 mm in thickness), and the assembly was placed on the jig of the LVDT.

The transducer was positioned in contact with the center of the coverslip and was fixed in place using two screws. A high-intensity light was irradiated using Bluephase N light-curing unit (Ivoclar Vivadent AG, Schaan, Liechtenstein, Germany) for 30 seconds from beneath the microscope slide. Upon light irradiation, the composite discs underwent shrinkage and a subsequent flexure, which were monitored by the transducer with the accuracy of 0.01 μm. The changes were recorded by a recorder on a computer. Since the shrinkage is mainly vertical in this method, only the changes in the thickness of the samples occurring within 30 minutes following the onset of light irradiation were recorded. One-way ANOVA and post-hoc Tukey’s test were used to compare the polymerization shrinkage at 1, 30, 60, and 1800 seconds following the onset of irradiation.

## RESULTS

Table 2 shows the mean polymerization shrinkage of the composite resins at 1, 30, 60, and 1800 seconds following the onset of irradiation. One-way ANOVA indicated a significant difference in the polymerization shrinkage of different composites at 1, 30, 60, and 1800 seconds (P<0.002). Thus, post-hoc Tukey’s honestly significant difference (HSD) test was applied for pairwise comparisons of the groups.

**Table 2.** Mean polymerization shrinkage (μm) of composite resins at 1, 30, 60, and 1800 seconds following the onset of irradiation

** Group **	** Composite **		** Minimum **	** Maximum **	** Mean **	** Std. Deviation **
** 1 **	** EXP **	1 s	0.37	1.49	0.85	0.48
		30 s	2.31	2.53	2.37	0.09
		60 s	2.46	2.70	2.53	0.09
		1800 s	2.84	3.27	3.03	0.15
		rate	0.40	0.76	0.61	0.13

** 2 **	** FBP **	1 s	0.07	0.29	0.19	0.07
		30 s	1.28	1.55	1.47	0.10
		60 s	1.47	1.73	1.66	0.10
		1800 s	1.97	2.28	2.15	0.12
		rate	0.19	0.27	0.24	0.03

** 3 **	** SF2 **	1 s	0.02	0.42	0.22	0.18
		30 s	1.32	1.45	1.39	0.04
		60 s	1.54	1.62	1.56	0.03
		1800 s	1.88	2.13	2.03	0.09
		rate	0.31	0.35	0.33	0.01

** 4 **	** TNB **	1 s	0.52	0.70	0.60	0.07
		30 s	1.46	1.64	1.55	0.07
		60 s	1.62	1.82	1.72	0.07
		1800 s	2.12	2.30	2.21	0.06
		rate	0.38	0.49	0.45	0.05

** 5 **	** XF **	1 s	0.07	0.55	0.38	0.18
		30 s	1.31	1.43	1.37	0.04
		60 s	1.44	1.56	1.50	0.05
		1800 s	1.80	1.98	1.87	0.07
		rate	0.31	0.43	0.36	0.05

** 6 **	** Z250 **	1 s	0.18	0.52	0.39	0.13
		30 s	1.40	1.51	1.47	0.04
		60 s	1.57	1.68	1.64	0.03
		1800 s	2.04	2.21	2.14	0.07
		rate	0.38	0.49	0.45	0.04

EXP=everX Posterior, FBP=Filtek Bulk-Fill Posterior, SF2=SonicFill 2, TNB=Tetric N-Ceram Bulk-Fill, XF=X-tra fil, Z250=Filtek Z250

The results of pairwise comparisons of the composite resins at 1 second after the onset of irradiation are presented in Table 3. As presented, at 1 second, EXP exhibited a significantly higher polymerization shrinkage compared to XF, SF2, and FBP (P<0.042). TNB and Z250 had no significant difference with the other groups. FBP showed the lowest polymerization shrinkage at 1 second (0.19±0.07 μm). Table 4 shows the results of pairwise comparisons of the composite resins at 30 seconds following the onset of irradiation.

**Table 3.** Pairwise comparisons of the polymerization shrinkage of composites at 1 second following the onset of irradiation

** 1 second **	** EXP **	** FBP **	** SF2 **	** TNB **	** XF **	** Z250 **
** EXP **	^ * ^					
** FBP **	P=0.002	^ * ^				
** SF2 **	P=0.004	P=1.000	^ * ^			
** TNB **	P=0.550	P=0.105	P=0.161	^ * ^		
** XF **	P=0.042	P=0.806	P=0.904	P=0.679	^ * ^	
** Z250 **	P=0.054	P=0.741	P=0.856	P=0.749	P=1.000	^ * ^

EXP=everX Posterior, FBP=Filtek Bulk-Fill Posterior, SF2=SonicFill 2, TNB=Tetric N-Ceram Bulk-Fill, XF=X-tra fil, Z250=Filtek Z250

**Table 4.** Pairwise comparisons of the polymerization shrinkage of composites at 30 seconds following the onset of irradiation

** 30 seconds **	** EXP **	** FBP **	** SF2 **	** TNB **	** XF **	** Z250 **
** EXP **	^ * ^					
** FBP **	P<0.001	^ * ^				
** SF2 **	P<0.001	P=0.499	^ * ^			
** TNB **	P<0.001	P=0.528	P=0.020	^ * ^		
** XF **	P<0.001	P=0.334	P=1.000	P=0.010	^ * ^	
** Z250 **	P<0.001	P=1.000	P=0.469	P=0.559	P=0.310	^ * ^

EXP=everX Posterior, FBP=Filtek Bulk-Fill Posterior, SF2=SonicFill 2, TNB=Tetric N-Ceram Bulk-Fill, XF=X-tra fil, Z250=Filtek Z250

As shown, EXP showed a significantly higher polymerization shrinkage compared to the other groups (P<0.001). TNB exhibited a significantly higher polymerization shrinkage than SF2 and XF (P<0.020). XF experienced the lowest polymerization shrinkage (1.37±0.04 μm) at this time point with an insignificant difference with SF2 (1.39±0.04 μm).

Table 5 shows the results of pairwise comparisons of the polymerization shrinkage of the composites at 60 seconds after the onset of light-curing. EXP had a significantly higher polymerization shrinkage than the other groups at this time point (P<0.001). TNB had a significantly higher polymerization shrinkage than SF2 and XF (P<0.026). FBP showed a significantly higher polymerization shrinkage than XF (P=0.023). XF exhibited the lowest polymerization shrinkage at this time point (1.50±0.05 μm).

**Table 5.** Pairwise comparisons of the polymerization shrinkage of composites at 60 seconds following the onset of irradiation

** 60 seconds **	** EXP **	** FBP **	** SF2 **	** TNB **	** XF **	** Z250 **
						
** EXP **	^ * ^					
** FBP **	P<0.00 1	^ * ^				
** SF2 **	P<0.00 1	P=0.306	^ * ^			
** TNB **	P<0.00 1	P=0.804	P=0.026	^ * ^		
** XF **	P<0.00 1	P=0.023	P=0.779	P=0.001	^ * ^	
** Z250 **	P<0.00 1	P=0.996	P=0.578	P=0.519	P=0.067	^ * ^

EXP=everX Posterior, FBP=Filtek Bulk-Fill Posterior, SF2=SonicFill 2, TNB=Tetric N-Ceram Bulk-Fill, XF=X-tra fil, Z250=Filtek Z250

Table 6 shows the results of pairwise comparisons of the polymerization shrinkage of the composites at 1800 seconds following the onset of irradiation. As shown, 30 minutes after the onset of polymerization (1800 seconds), EXP showed a significantly higher polymerization shrinkage than the other groups (P<0.001).

**Table 6.** Pairwise comparisons of the polymerization shrinkage of composites at 1800 seconds following the onset of irradiation

** 1800 seconds **	** EXP **	** FBP **	** SF2 **	** TNB **	** XF **	** Z250 **
** EXP **	^ * ^					
** FBP **	P<0.001	^ * ^				
** SF2 **	P<0.001	P=0.469	^ * ^			
** TNB **	P<0.001	P=0.944	P=0.108	^ * ^		
** XF **	P<0.001	P=0.003	P=0.170	P<0.001	^ * ^	
** Z250 **	P<0.001	P=1.000	P=0.532	P=0.914	P=0.004	^ * ^

EXP=everX Posterior, FBP=Filtek Bulk-Fill Posterior, SF2=SonicFill 2, TNB=Tetric N-Ceram Bulk-Fill, XF=X-tra fil, Z250=Filtek Z250

TNB, FBP, and Z250 experienced a significantly higher polymerization shrinkage compared to XF (P<0.004). XF showed the lowest polymerization shrinkage at this time point (1.87±0.07 μm).

Moreover, EXP showed a significantly higher speed of polymerization compared to the other groups (P<0.010). TNB and Z250 showed a significantly higher speed of polymerization compared to FBP as well (P=0.001). The lowest polymerization speed was noted with FBP (0.24±0.03 μm).

Table 7 shows the results of Tukey’s test in comparing the shrinkage strain rate of the composite resins.

**Table 7.** Comparison of the shrinkage strain rate of composite resins

** Rate **	** EXP **	** FBP **	** SF2 **	** TNB **	** XF **	** Z250 **
** EXP **	^ * ^					
** FBP **	P<0.001	^ * ^				
** SF2 **	P<0.001	P=0.347	^ * ^			
** TNB **	P=0.010	P=0.001	P=0.106	^ * ^		
** XF **	P<0.001	P=0.106	P=0.982	P=0.347	^ * ^	
** Z250 **	P=0.010	P=0.001	P=0.106	P=1.000	P=0.347	^ * ^

EXP=everX Posterior, FBP=Filtek Bulk-Fill Posterior, SF2=SonicFill 2, TNB=Tetric N-Ceram Bulk-Fill, XF=X-tra fil, Z250=Filtek Z250

**Fig. 2.**
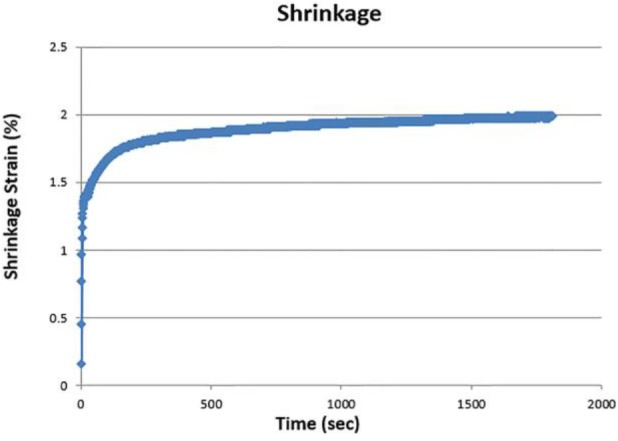
Shrinkage strain of composite resins

**Fig. 3.**
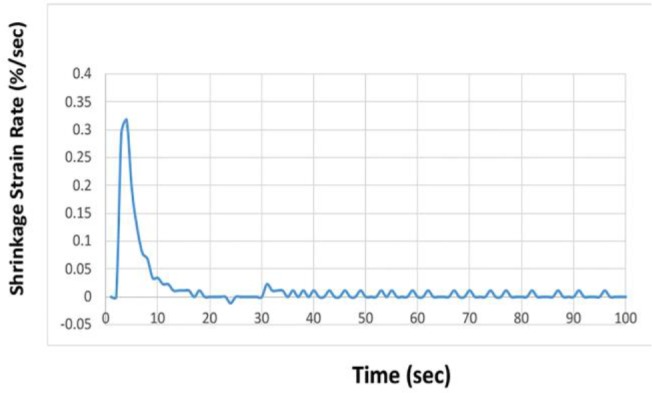
Shrinkage strain rate of composite resins

Figures 2 and 3 illustrate the shrinkage strain and shrinkage strain rate of the composite resins evaluated in the current study.

## DISCUSSION

The present study assessed the polymerization shrinkage of five bulk-fill composites in comparison with a conventional composite. The groups were significantly different regarding polymerization shrinkage (P<0.002). The polymerization shrinkage of the tested composites following the onset of light irradiation ranged from 0.19 to 3.03. EXP showed a significantly higher polymerization shrinkage than the other composites at 30, 60, and 1800 seconds after the onset of light irradiation, while XF experienced the lowest polymerization shrinkage at the aforementioned time points.

The bonded disc technique was used to quantify the changes in the height of the composite samples, which indicate their polymerization shrinkage during and after light-curing. The main advantage of this technique is that it enables a fast assessment of polymerization shrinkage and allows the use of different intensities of light, especially at different temperatures [13,14]. In this technique, the volumetric shrinkage is estimated according to the axial shrinkage, and the C-factor of the samples should be >5 in order to be able to evaluate the conditions causing the highest polymerization shrinkage in the clinical setting. However, the consistency of the composite resin is also important in this respect [13]. In this method, upon light irradiation, the composite disc undergoes flexion as a result of shrinkage, which is recorded by the transducer of the device; the volumetric shrinkage of the sample is determined as such.

The volumetric shrinkage of composite resins depends on factors such as the amount, type, and size of fillers [15]. In general, increasing the number of fillers in the resin matrix decreases the overall shrinkage of composite resins due to the reduced amount of monomers available for the curing reaction. However, it can also increase the elastic modulus of the material and lead to a high shrinkage stress [15,16].

On the other hand, it has been suggested that the addition of great amounts of fillers to decrease the resin volume is not an efficient approach to decrease the polymerization shrinkage and stress. Thus, chemical modification is another adopted approach to slow down the polymerization rate and to decrease the polymerization shrinkage stress [17]. Other factors affecting polymerization shrinkage include the type of resin matrix, the concentration of monomers, and the type of the initiators as they determine the polymer structure of composite resins [18]. Moreover, all factors controlling the degree of conversion also affect polymerization shrinkage, including the reactivity of monomers and cross-linking [19,20]. For example, triethylene glycol dimethacrylate (TEGDMA), which is present in the composition of highly flowable restorative materials, has a high reactivity and results in a higher conversion of double bonds and consequently a higher shrinkage [21–24].

The magnitude of the volumetric shrinkage and the amount of generated stress during the polymerization reaction of composite resins are the main factors causing poor marginal adaptation, postoperative pain, and secondary caries in vivo [19].

Tsujimoto et al [25] showed that volumetric shrinkage started immediately after the initiation of light irradiation and continued even after its discontinuation. Continuation of shrinkage after the completion of light irradiation may be due to the post-polymerization reaction of residual monomers. Yu et al [26] stated that the mean shrinkage of bulk-fill composite resins ranges from 1.5% to 3.4%, while this range is 2.1% to 4.3% for conventional composites. The shrinkage rate of composite resins evaluated in our study was within the range for conventional composite resins. In the study by Jang et al [27], TNB showed the minimum polymerization shrinkage stress. Several factors may affect the results in this respect. These composites contain a shrinkage stress reliever, which is a filler functionalized with saline. It has a lower modulus of elasticity, and therefore, acts as a microscopic spring, neutralizing the forces generated during shrinkage. These composites also contain pre-polymerized fillers. Composite resins containing pre-polymerized fillers often show a relatively low modulus of elasticity [27,28].

Our findings regarding the higher polymerization shrinkage of EXP are in agreement with those reported by Fronza et al [29]. They showed that despite having a high percentage of debonded areas, EXP has a relatively small marginal gap (about 15 μm). It is believed that during polymerization, composites cannot contract along the long fibers in their composition; consequently, they preserve their original horizontal dimensions although the resin matrix tries to contract vertically [29]. This behavior has not been seen with TNB, despite its high mineral content. In fact, this composite showed less polymerization shrinkage stress and a potential for marginal gap formation in the study by Fronza et al [29], which may be due to the presence of stress relievers in its composition [30].

In the current study, at 1 second (the initiation of polymerization), FBP showed the lowest polymerization shrinkage; it showed the lowest polymerization rate as well. In fact, the manufacturers claim that FBP has novel monomers that act to decrease the polymerization stress [31]. In our study, at all time points, except for 1 second, XF showed the lowest shrinkage. According to the manufacturer, this composite is a combination of a multi-hybrid filler and a novel initiator to minimize the polymerization shrinkage [11]. At 1 second, FBP showed the lowest shrinkage. It appears that polymerization reactions did not sufficiently proceed in this composite. In the composite resins evaluated in our study, the shrinkage curve raised immediately after the onset of photo-activation, and the highest rate of shrinkage was noted early after the initiation of light irradiation; the shrinkage gradually increased afterwards. Bulk-fill composites did not have a significant difference with the conventional composite (the control group) in terms of polymerization shrinkage and polymerization rate at 1 second (the initiation of polymerization). Our study had an in-vitro design. Clinical conditions cannot be accurately simulated in vitro. Thus, the generalization of the results to the clinical setting must be done with caution. In the assessment of the polymerization rate, the highest rate was noted for EXP, and the lowest rate was recorded with XF. Other composites had no significant difference with the control group. Since the composite resins used in the current study are all patented, the energy dispersive X-ray (EDX) analysis is recommended to find the composition of these products. Also, future studies are required to assess the wear resistance and fracture toughness of bulk-fill composites in comparison with conventional composites.

## CONCLUSION

Within the limitations of the present in-vitro study, the results showed that the polymerization shrinkage of the evaluated bulk-fill composites was not significantly different from that of the conventional composite. XF showed the lowest polymerization shrinkage among the bulk-fill composites.
